# Supplementary material for: A sensory memory processing system with multi-wavelength synaptic-polychromatic light emission for multi-modal information recognition
Source: Nat Commun. 2023 May 8;14:2648. doi: 10.1038/s41467-023-38396-7 (PMC10167252; doi:10.1038/s41467-023-38396-7)
Supplement: Supplementary file 1 — SUPPLEMENTARY INFO [file 41467_2023_38396_MOESM1_ESM.pdf]

Supplementary Information

A sensory memory processing system with multi-wavelength synaptic-polychromatic light emission for multi-modal information recognition

Liuting Shan[1,2], Qizhen Chen[1,2,3], Rengjian Yu[1,2], Changsong Gao[1,2], Lujian Liu[1,2], Tailiang Guo[1,2], Huipeng Chen[1,2]*

[1]Institute of Optoelectronic Display, National & Local United Engineering Lab of Flat Panel Display Technology, Fuzhou University, Fuzhou 350002, China

[2]Fujian Science & Technology Innovation Laboratory for Optoelectronic Information of China, Fuzhou 350100, China

[3] School of Opto-electronic and Communication Engineering, Xiamen University of Technology, Xiamen 361024, China

Email: hpchen@fzu.edu.cn

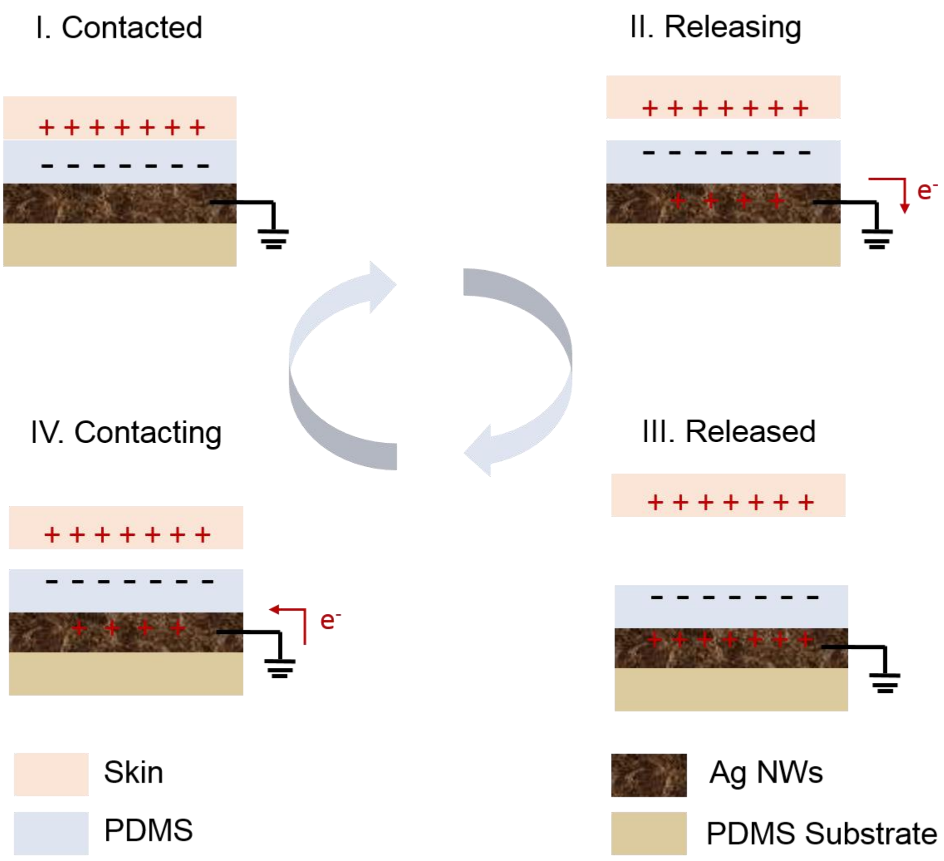

Supplementary Fig. S1. Working schematic of the single-electrode TENG. In contact with human skin, due to different electron affinity, the skin and the PDMS friction layer generate equal amount of positive and negative charges respectively. Electrons conduct from skin to PDMS surface due to triboelectric effect (Fig. S1, I). When the skin is separated from PDMS, the charges are unbalanced. In order to neutralize the charges, Ag NWs electrode begins to charge positively, and electrons flow through the ground (Fig. S1, II). When the skin is far enough away from the TENG, the charges reach equilibrium (Fig. S1, III). When the skin is close to PDMS again, electrons flow back to Ag NWs electrode, neutralizing the positive charges of Ag NWs until the skin is full contact with PDMS (Fig. S1, IV). In this way, circulating signals will be generated on the electrode.

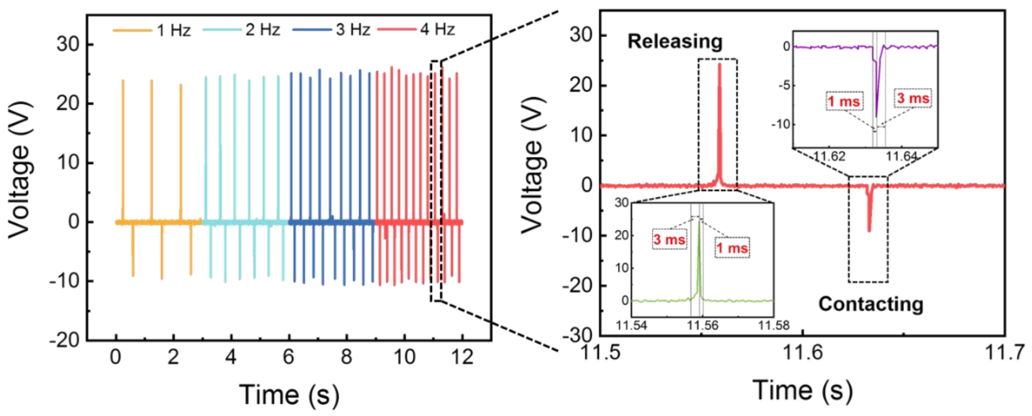

Supplementary Fig. S2. The open-circuit voltage frequency output of the TENG. The open-circuit voltage when the frequency from 1 Hz to 4 Hz when the pressure is 6 kPa and one working cycle of the TENG. The rise time and fall time of the releasing signal are 3 ms and 1 ms and the rise and fall of the contacting signal are 1ms and 3ms, respectively.

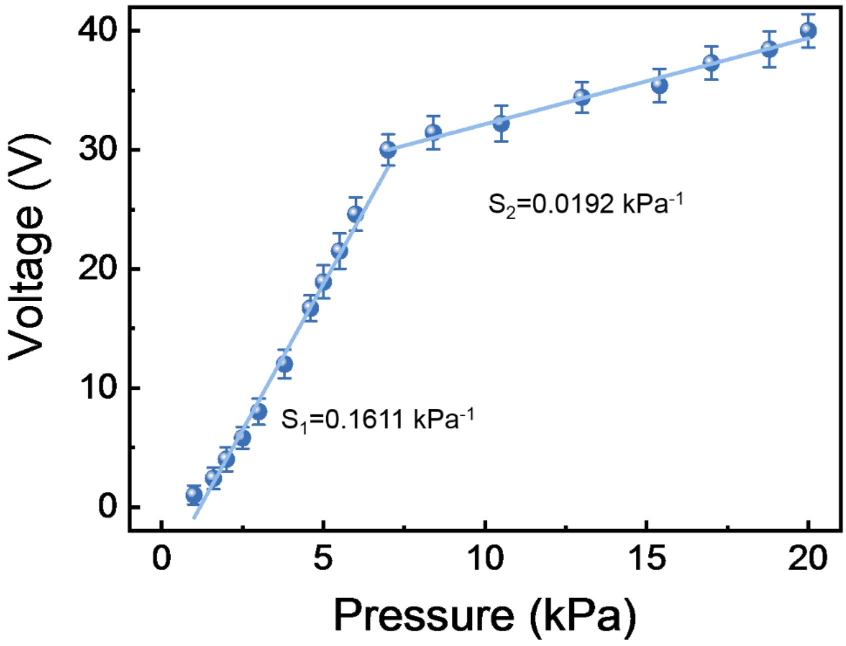

Supplementary Fig. S3. The sensitivity of TENG. The pressure sensitivity (S) is calculated by the formula: $S = (\Delta V/Vmax)/\Delta P$. Here, ΔV is the open-circuit voltage (V_{OC}) variation, V_{max} is the maximum open-circuit voltage, ΔP is the pressure variation. The error bars in the figure represent the standard deviation (SD). It should be noted that this curve shows two different regions. In the low-pressure region (<7 kPa), TENG shows a well-behaved linear response with a pressure sensitivity of 0.1611 kPa^{-1}, while in the high-pressure region (>7 kPa), the pressure sensitivity is 0.0192 kPa^{-1}.

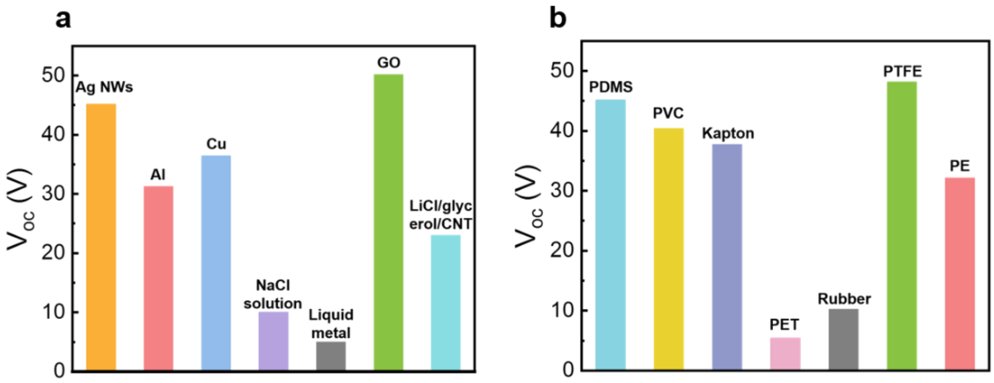

Supplementary Fig. S4. The performance comparison of TENG. (a) Different electrode materials or **(b)** Different friction layer materials. CNT: carbon nanotube; GO: graphene oxide; PVC: Polyvinyl Chloride; PDMS: Polydimethylsiloxane; Kapton: Polyimide; PE: Polyethylene; PET: Polyethylene Terephthalate.

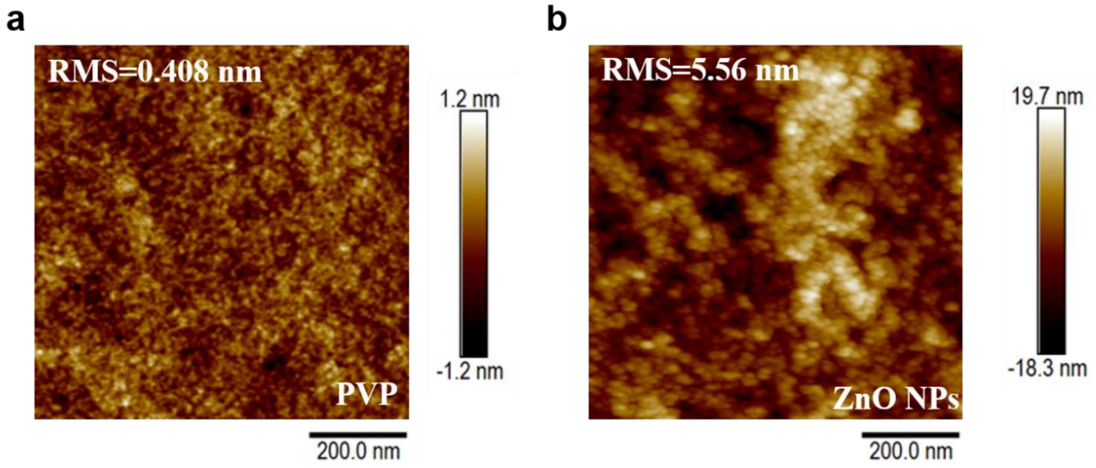

Supplementary Fig. S5. The AFM image of PVP film and ZnO NPs films. The PVP film and ZnO NPs film show the root-mean-square roughness of 0.408 nm and 5.56 nm, respectively.

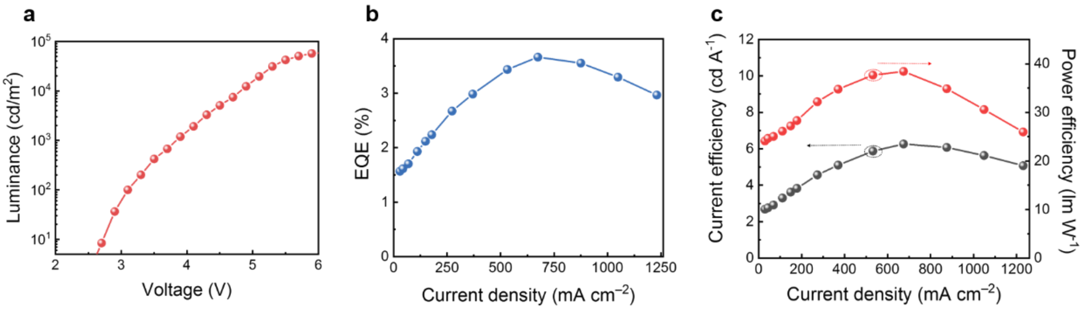

Supplementary Fig. S6. The device performances of QLED. (a) The voltage-luminance characteristic of the QLED under forward-bias. **(b)** EQE of the devices as a function of current density. **(c)** CE and PE of the devices as a function of current density.

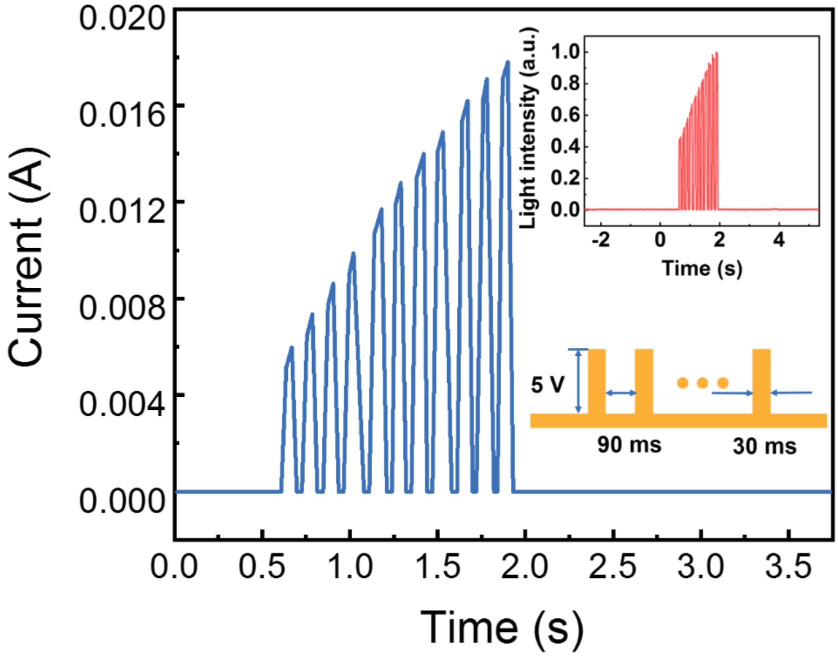

Supplementary Fig. S7. The electrical output behavior of synaptic device under the stimulation of circulating electrical pulses (5 V amplitude, 30 ms duration, 90 ms interval, the inset shows the corresponding eleven light output pulses).

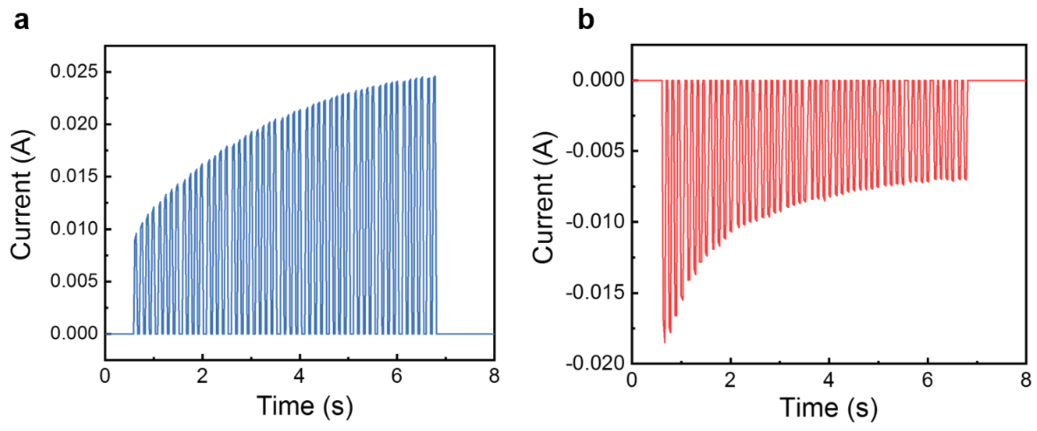

Supplementary Fig. S8. Current as a function of time under positive pulses (5 V amplitude, 30 ms duration, 90 ms interval) and negative pulses (-5 V amplitude, 30 ms duration, 90 ms interval), which are equivalent to the potentiation and the depression processes, respectively.

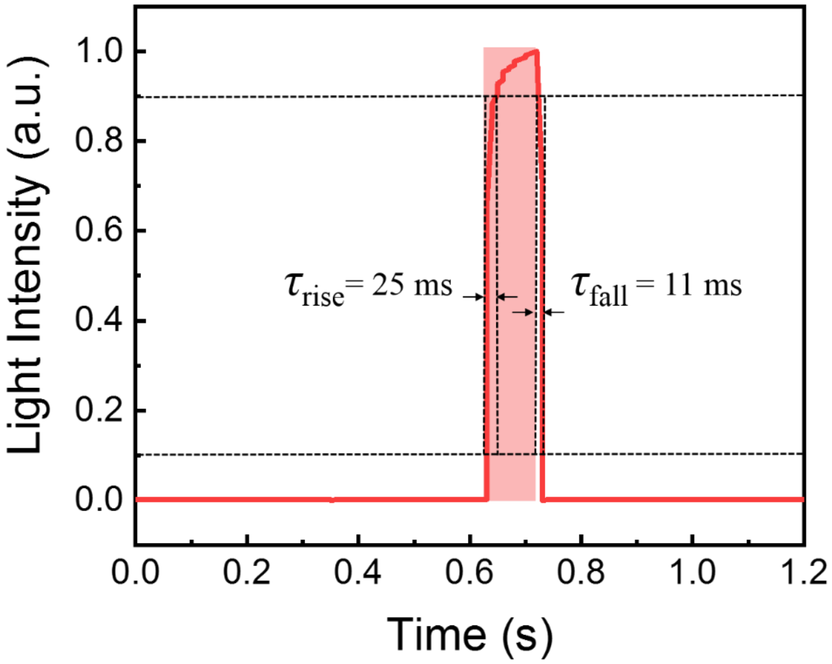

Supplementary Fig. S9. The light output time response of the QLED. The response time is defined as the time difference between 10% and 90% of the maximum EPSB. The rise (τ_{rise}) and fall (τ_{fall}) times of QLED are also marked in the figure. When a square wave pulse bias at V_{in} (6 V amplitude, 90 ms pulse width) is used, the rise and fall times are τ_{rise} ~25 ms and τ_{fall} ~11 ms.

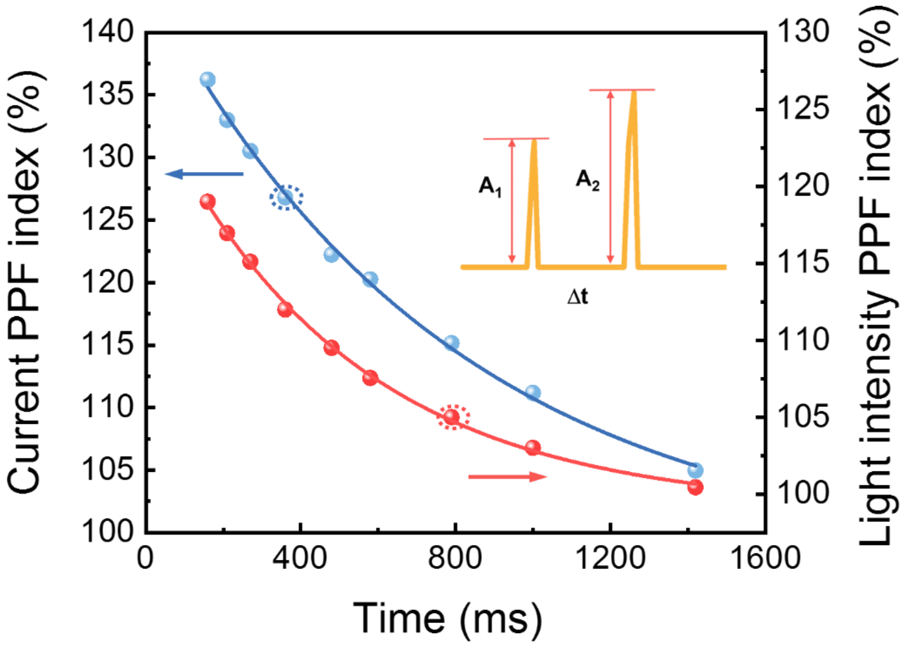

Supplementary Fig. S10. Current and light PPF index as a function of the presynaptic spike internal Δt. The maximum current PPF index is 136% at Δt value of 160 ms. With the increase of Δt value, the current PPF index gradually decreases to about 104%; the maximum light intensity PPF index is 119% at Δt value of 160 ms. With the increase of Δt value, the light intensity PPF index gradually decreases to about 101%. The continuous line is the result of fitting using a single exponential decay function. Here, PPF index is defined as $A_2/A_1 \times 100$ %, where A_1 and A_2 are the peaks of the first and second spike respectively.

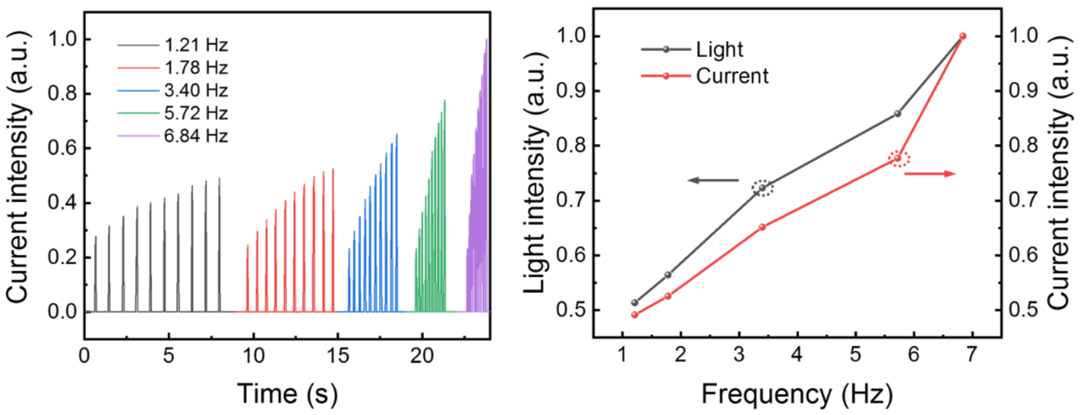

Supplementary Fig. S11. The frequency response of EPSC and the maximum frequency response of EPSC and EPSB from 1.21 Hz to 6.84 Hz (6 V amplitude, 30 ms duration).

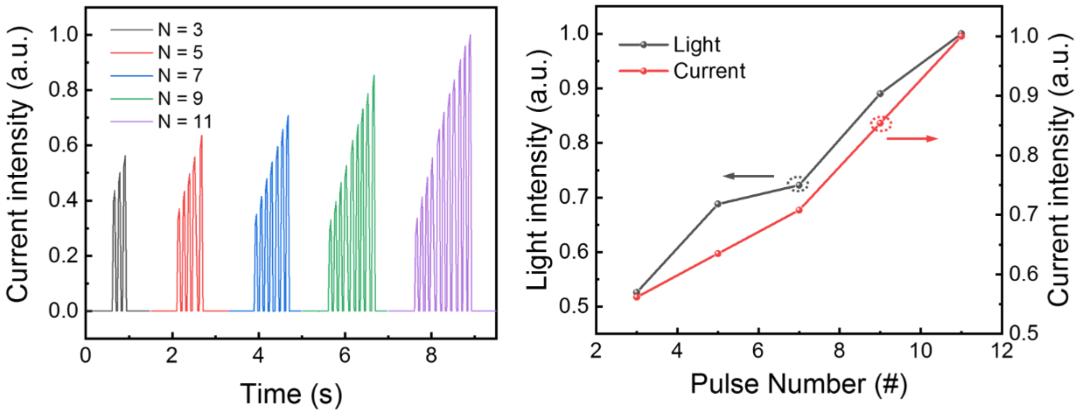

Supplementary Fig. S12. The number response of EPSC and the maximum number response of EPSC and EPSB from 3 to 11 pulses (6 V amplitude, 30 ms duration).

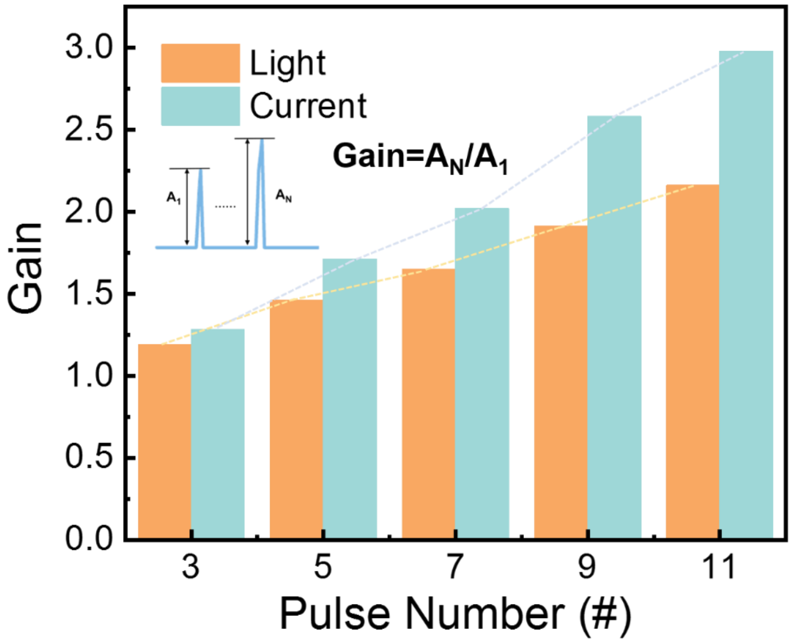

Supplementary Fig. S13. EPSB and EPSC gain (A_N/A_1) (determined by the ratio of the Nth EPSC peak (A_N) to the first EPSC peak (A_1)) of the artificial synapse from N=3 to N=11 (6 V amplitude, 30 ms duration). EPSC and EPSB gains vary from 1.28 to 2.97 and 1.19 to 2.16, respectively.

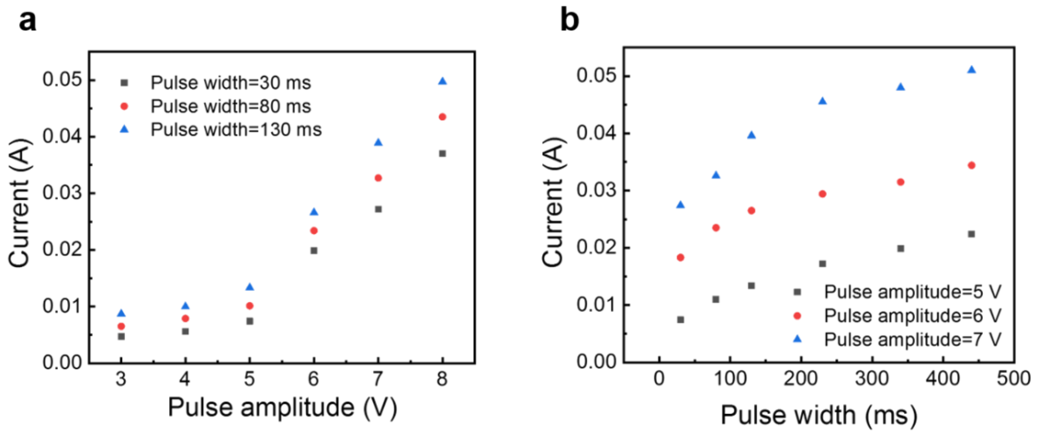

Supplementary Fig. S14. Amplitude current response and pulse width current response characteristics of device. The modulation of postsynaptic electric signal by changing the width and amplitude of the pulse.

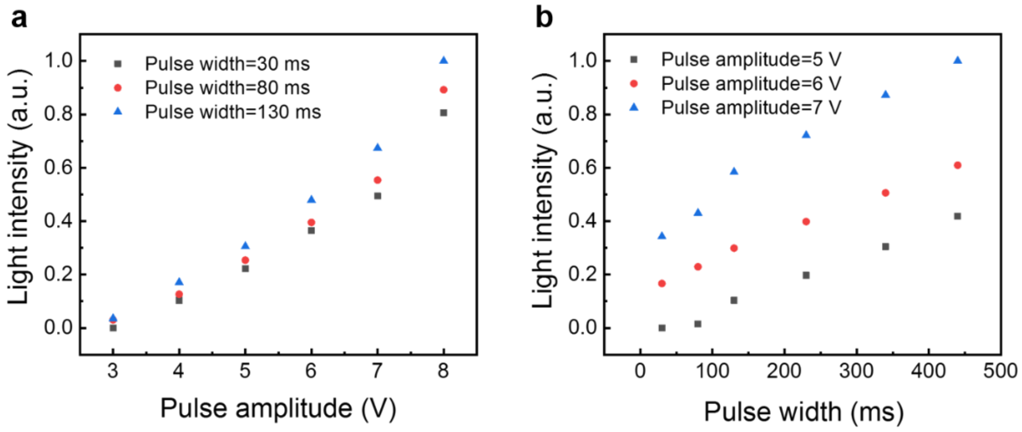

Supplementary Fig. S15. Amplitude light response and pulse width light response characteristics of device. The modulation of postsynaptic light signal by changing the width and amplitude of the pulse.

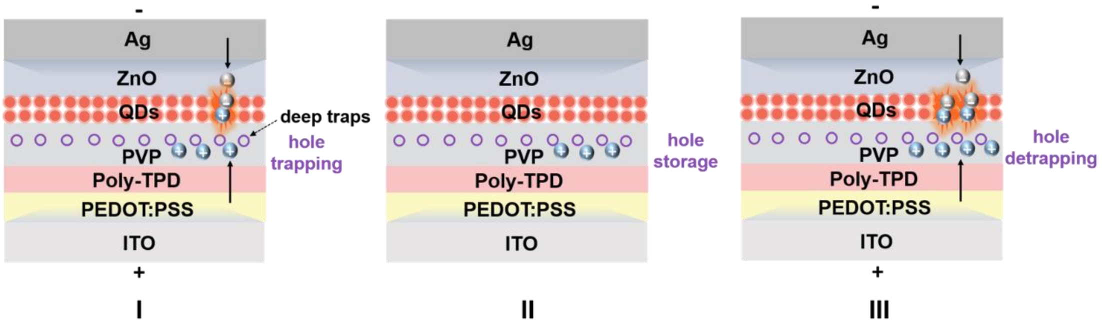

Supplementary Fig. S16. The working mechanism of PVP charge trapping layer. The blue balls represent the holes and the gray ones represent the electrons. The purple circles represent deep traps. Part of the holes can be captured by the PVP layer when bias is applied (I). Then, when the bias is removed, the holes are stored in the PVP layer (II). When bias is applied again, trapped holes are released under the action of an applied electric field (III), thus increasing the conductivity and brightness of the device and achieving a simulation of synaptic plasticity.

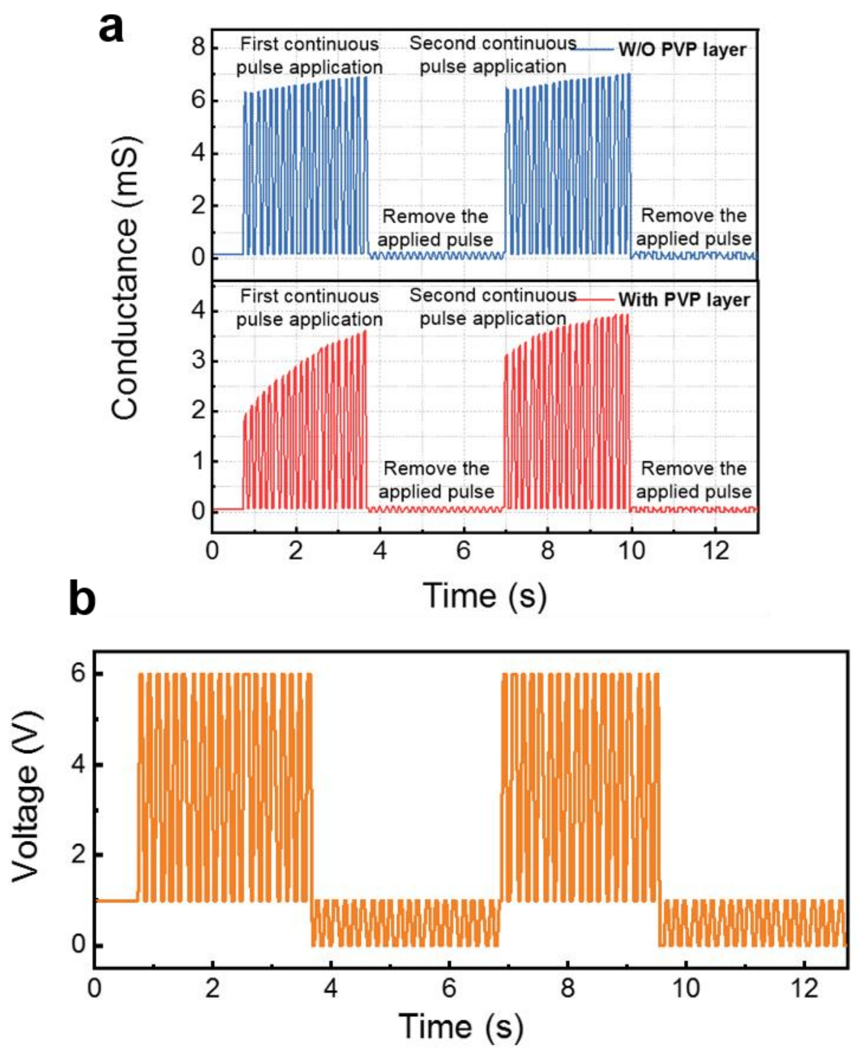

Supplementary Fig. 17. Conductance of a QLED under multiple write-erase pulses. The write pulse stimulation continuously increases the device current, and after the erase pulse is applied, the device conductance can return to its original state. (**a**) Conductance change between devices without PVP layer and devices with PVP layer. (**b**) The waveform of the applied pulse. The write pulse stimulation continuously increases the device conductance and the conductance of the device with PVP layer changes significantly than that of the device without PVP layer even after the application of the erase pulse.

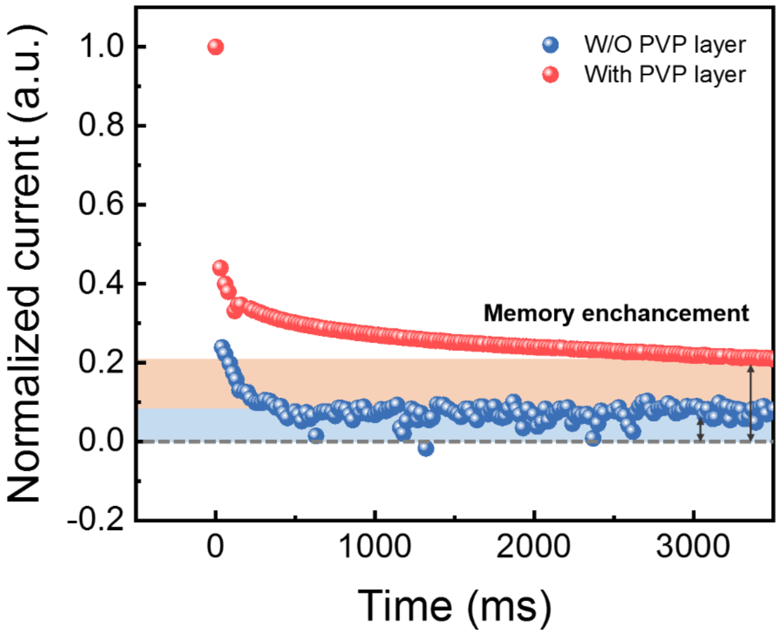

Supplementary Fig. 18. Long-term decay time curves of devices without and with PVP layer (the ordinate is normalized by the log of the current). The shaded area is the memory enhancement of the respective device relative to the initial resting current level.

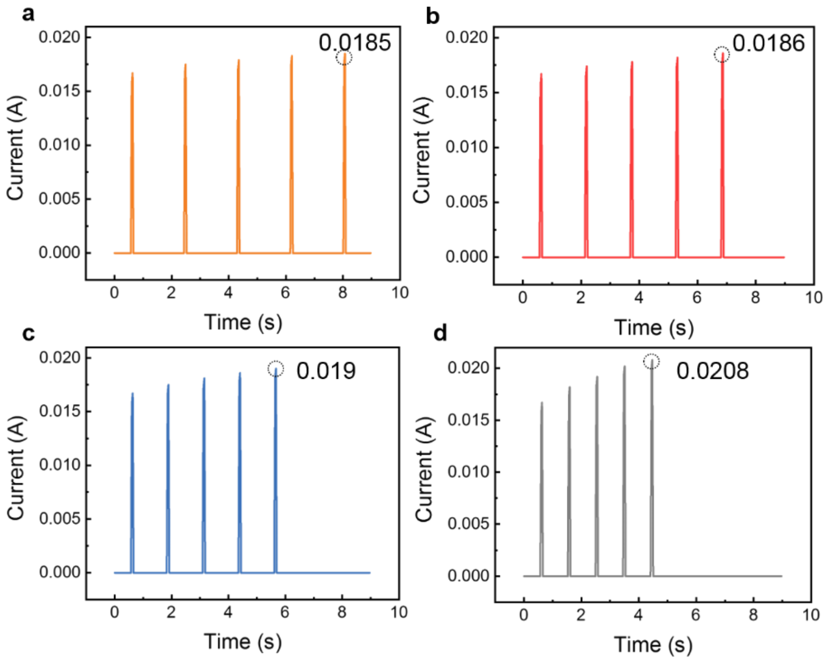

Supplementary Fig. 19. Postsynaptic current in final state at different frequencies. Under different frequencies from a to d, corresponding to the step frequency change from low to high. Light-emitting synaptic device can generate a response proportional to the frequency change.

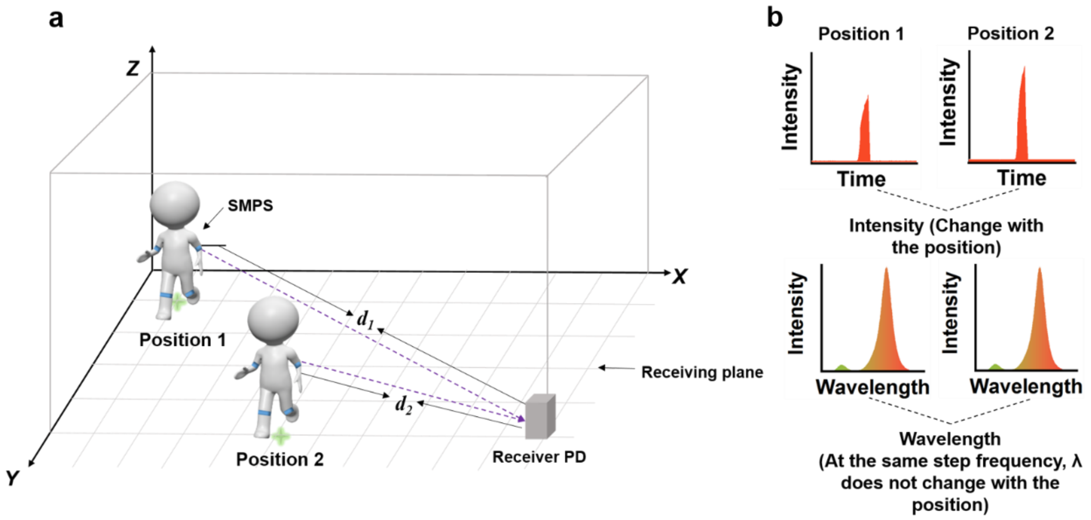

Supplementary Fig. 20. The change of the output light intensity and wavelength with the position. When the motion amplitude is fixed, the output light intensity increases as the distance between human and the detector decreases, and the wavelength of light is determined at the time of emission, so it is not affected by distance.

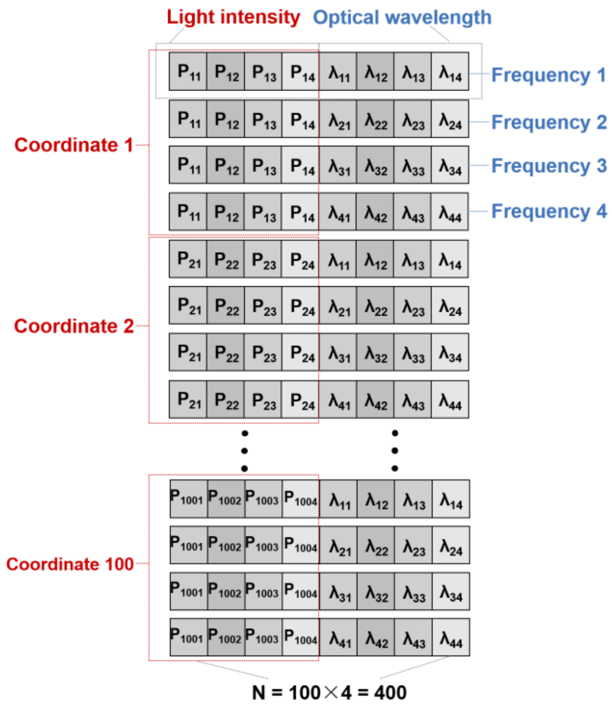

Supplementary Fig. 21. Data set composed of light intensity and wavelength vector.

Database of light intensity and wavelength through the measured data of 100×4 sets.
